# A potentially functional variant of *ARID1B* interacts with physical activity in association with risk of hepatocellular carcinoma

**DOI:** 10.18632/oncotarget.16074

**Published:** 2017-03-10

**Authors:** Li Liu, Nana Tian, Chengyu Zhou, Xinqi Lin, Weibiao Lv, Zhifeng Lin, Zibo Lin, Yongfen Qi, Yi Yang, Sidong Chen, Xinfa Yu, Yanhui Gao

**Affiliations:** ^1^ Department of Epidemiology and Biostatistics, School of Public Health, Guangdong Pharmaceutical University, Guangzhou, China; ^2^ Department of Oncology, Shunde First People's Hospital, Foshan, China; ^3^ Department of Clinical Laboratory, Shunde First People's Hospital, Foshan, China

**Keywords:** ARID1B, genetic variant, functional annotation, gene-environment interaction, hepatocellular carcinoma

## Abstract

The tumor suppressor role of AT-rich interactive domain containing protein 1B (*ARID1B*) has drawn much attention in area of cancer etiology. However, it had remained unknown whether or not genetic variants of *ARID1B* involved in development of hepatocellular carcinoma (HCC). In this study, three putatively functional variants in *ARID1B* (rs73013281C>T, rs167007A>G, and rs9397984C>T) were selected using bioinformatics tools, and a case-control study of 611 cases and 614 controls was conducted to investigate genetic associations with HCC risk in a Southern Chinese population. Two-dimensional gene-environment interactions were also explored using both multiplicative and additive scales. A dominant effect of the rs73013281 was found for HCC risk, with an adjusted odds ratio (OR) of 1.70 [95% confidence interval (CI) = 1.03−2.80] for the CT/TT genotypes compared to the CC genotype. In stratified analysis, the detrimental effect of the T allele on elevated HCC risk was attenuated by physical activity, with an adjusted OR of 2.75 (95% CI = 1.39−5.41) among inactive individuals against that of 0.89 (95% CI = 0.42−1.91) in those who exercised regularly. Expectably, the rs73013281 showed both multiplicative and additive interactions with physical activity (*P* = 0.037 and 0.006, respectively). In conclusion, these results highlighted the significant genetic contribution of the *ARID1B* variant, rs73013281, to susceptibility for HCC, especially in interaction with physical activity.

## INTRODUCTION

The perturbation of SWItch/Sucrose NonFermentable (SWI/SNF) complexes is an emerging theme in carcinogenesis. The SWI/SNF complexes, as one family of ATP-dependent chromatin remodelers, have essential role in control of gene expression and thus regulate critical cellular processes, including cell proliferation, differentiation, apoptosis, and DNA repair [1]. Sequencing and biochemical evidence have suggested the link of these complexes to various cancers, as somatic mutations that lead to abnormal function of their subunits have been identified at high frequencies in at least 20% of malignancies [2–8].

The largest subunit in SWI/SNF, named AT-rich interactive domain containing protein 1B (ARID1B), has been recently characterized to play tumor suppressor activity [5, 9]. The *ARID1B* gene, located at 6q25.1, encodes this core subunit, which executes DNA binding function without sequence specificity [10]. Importantly, multiple studies utilizing next-generation sequencing technology have revealed that *ARID1B* was frequently mutated in a subset of cancers, including prostate, gastric, colorectal and liver cancers [2, 7, 11, 12], among which, a recent whole-genome sequencing (WGS) analysis of hepatocellular carcinoma (HCC) has listed *ARID1B* as one of recurrently mutated genes [2]. In the WGS set, missense mutations and deletion of *ARID1B* were detected in 3 out of 27 tumors (11.1%), while in the validation set with 120 HCCs, 8 additional mutations of *ARID1B* (6.7%) were identified by exon sequencing. Moreover, prominent growth promoting effect was observed for the knockdown of *ARID1B* in two HCC cell lines [2]. The aberrations of ARID1B could destabilize the function of SWI/SNF complexes in regulation of gene expression, especially anti-oncogenic and oncogenic pathways [13, 14], and consequently might involve in hepatocellular carcinogenesis.

HCC is the third leading cause of cancer-related death globally, affecting all the world populations [15]. More than 80% of cases occurred in sub-Saharan Africa and Asia, especially in China [16]. But be warned, the incidence of HCC showed an upward trend in western countries in recent years [15]. Several major risk factors have been established for HCC, including chronic infection with hepatitis B (HBV) and hepatitis C viruses (HCV), chronic alcohol consumption, and dietary exposure to mycotoxin aflatoxins [17]. However, in individuals exposed to these risk factors, only a fraction eventually would develop to HCC during their lifespan, implicating a strong genetic component to the inter-individual variation in HCC susceptibility. The exploration of genetic susceptibility factors would provide valuable assistance to prediction of cancer risk and early detection of HCC.

Notably, accumulating evidence has drawn attention to the tumor suppressor role of *ARID1B* for HCC as stated above. The effect of genetic variants in *ARID1B* for HCC development, however, has never been investigated. Therefore, we initially performed functional annotations of *ARID1B* variants using the bioinformatics tools, HaploReg V4.1 [18] and PolyPhen-2 [19], and then conducted a case-control study, including 611 cases and 614 controls, to explore the effect of three *ARID1B* potentially functional variants (rs73013281C>T, rs167007A>G, and rs9397984C>T) and their interaction with lifestyle on HCC incidence in a Southern Chinese population.

## RESULTS

### Subjects’ characteristics

The characteristics of study subjects are presented in Table 1. No significant differences were observed between cases and controls in the distribution of age and gender, suggesting the frequency matching was adequate. As expected, HBV infection was considered as the most important risk factor for HCC, with an adjusted OR of 14.48 (95% CI = 10.77–19.47; Supplementary Table 1). Smoking, drinking, and family history of HCC were also significantly associated with increased risk of HCC, while the non-occupational physical activity conferred decreased risk of HCC. Therefore, as potential confounders, age, gender, and these influence factors for HCC were adjusted for in the following analysis of genetic associations.

**Table 1:** Characteristics of HCC cases and controls in this study

Variables	Cases (%) *N* = 611	Controls (%) *N* = 614	χ^2^	*P*
Age (y), mean ± SD	55.35 ± 11.81	55.93 ± 12.10		0.397^a^
Gender			0.15	0.699
Males	532 (86.9)	530 (86.3)		
Females	79 (13.1)	84 (13.7)		
Smoking status			17.49	< 0.001
Never	194 (31.8)	266 (43.3)		
Ever	417 (68.2)	348 (56.7)		
Drinking status			15.78	< 0.001
Never	268 (43.9)	339 (55.2)		
Ever	343 (56.1)	275 (44.8)		
Physical activity status			16.33	< 0.001
Inactive	391 (64.0)	323 (52.6)		
Active	220 (36.0)	291 (47.4)		
HBsAg status			399.11	< 0.001
Negative	157 (25.7)	507 (82.6)		
Positive	454 (74.3)	107 (17.4)		
HCC Family history			39.37	< 0.001
No	526 (86.1)	591 (96.3)		
Yes	85 (13.9)	23 (3.7)		

Abbreviations: HCC, hepatocellular carcinoma; SD, standard deviation.

^a^*P* value was calculated by the *t* test.

### Associations between individual variants and HCC risk

Genotypic distribution of the three potentially functional variants in *ARID1B* (rs73013281, rs167007, and rs9397984) among cases and controls are shown in Table 2. All the variants conformed to Hardy-Weinberg equilibrium in controls (Supplementary Table 2). Multivariate logistic regression model showed that, as compared to the CC genotype, the rs73013281TT genotype had an OR of 1.74 for HCC risk (95% CI = 1.04–2.92), while the CT genotype showed a marginally significant association with the risk (OR = 1.63, 95% CI = 0.96–2.77; Table 3). After the multiple comparison correction by a permutation test, the TT genotype retained its significant association with HCC risk (*P* for permutation test = 0.048). A dominant effect of the rs73013281 was found for HCC risk, with an adjusted OR of 1.70 (95% CI = 1.03–2.80; *P* for permutation test = 0.047). No significant associations were seen between other variants and HCC risk.

**Table 2:** Differences in genotypic distribution of the *ARID1B* variants between cases and controls

Genotypes	Cases (%) *N* = 611	Controls (%) *N* = 614	χ^2^	*P*	*P*_perm_^a^
rs73013281			5.14	0.077	0.080
CC	44 (7.2)	67 (10.9)			
CT	240 (39.4)	236 (38.5)			
TT	325 (53.4)	310 (50.6)			
rs167007			1.10	0.578	0.593
AA	6 (1.0)	3 (0.5)			
AG	83 (13.6)	87 (14.3)			
GG	522 (85.4)	520 (85.2)			
rs9397984			1.24	0.537	0.545
CC	4 (0.6)	2 (0.3)			
CT	70 (11.5)	79 (12.9)			
TT	536 (87.9)	530 (86.7)			

^a^*P*_perm_ values for χ^2^ test were calculated by the permutation method with 10,000 repeats.

**Table 3:** Genetic associations between *ARID1B* variants and risk of hepatocellular carcinoma

Genotypes	Crude OR (95% CI)	*P*	*P*_perm_^a^	Adjusted OR (95% CI)^b^	*P*^b^	*P*_perm_^c^
rs73013281						
CC	1.00			1.00		
CT	1.55 (1.02–2.36)	0.042	0.060	1.63 (0.96–2.77)	0.071	0.092
TT	1.60 (1.06–2.41)	0.026	0.045	1.74 (1.04–2.92)	0.036	0.048
CT+TT	1.58 (1.06–2.35)	0.025	0.034	1.70 (1.03–2.80)	0.040	0.047
rs167007						
AA+AG	1.00			1.00		
GG	1.02 (0.74–1.39)	0.926	0.935	0.84 (0.57–1.23)	0.364	0.378
rs9397984						
CC+CT	1.00			1.00		
TT	1.11 (0.79–1.55)	0.555	0.619	0.93 (0.62–1.41)	0.745	0.777

Abbreviations: OR, odds ratio; 95% CI, 95% confidence interval.

^a^*P*_perm_ values were calculated by the permutation method with 10,000 repeats.

^b^ORs, 95% CIs, and *P* values were calculated by logistic regression analysis after adjusting for age, gender, smoking and drinking status, physical activity, HBV infection status, and family history of HCC.

^c^*P*_perm_ values were calculated using the permutation method under the multivariate logistic regression model after adjusting for age, gender, smoking and drinking status, physical activity, HBV infection status, and family history of HCC.

We further performed haplotypes analysis of the variants in *ARID1B* (in the order of rs73013281, rs167007, and rs9397984; Supplementary Table 3). These three variants were in high linkage disequilibrium, all D’ > 0.84, with each other. Three common haplotypes were observed (frequency > 5%), and no associations were detected with the disease risk.

### Associations between gene-environment interaction and HCC risk

Table 4 describes the results of stratified analysis and pair-wise gene-environment interaction analysis of the promising variant, rs73013281. When stratified by the non-occupational physical activity, the inactive individuals with the T allele yielded an OR of 2.75 (95% CI = 1.39–5.41) compared to those carrying the CC genotype, whereas the effect of the T allele on increased risk was significant attenuated in the individuals who exercised regularly (OR = 0.89; 95% CI = 0.42–1.91). Expectably, the rs73013281 showed both significant multiplicative and additive interactions with physical activity in HCC susceptibility (*P* = 0.037 and 0.006 for multiplicative and additive interactions, respectively). We also performed the interaction analysis of this variant with other risk factors for HCC, including smoking and drinking status, HBV infection status, and family history of HCC. However, no significant interactions were seen between the rs73013281 and these HCC risk factors.

**Table 4:** Pair-wise interaction of rs73013281 with risk factors on HCC risk

Stratified variables	Genotypes	Cases/Controls	OR (95% CI)^a^	*P*_mult_^b^	*P*_add_^c^
Smoking status				0.273	0.060
Never	CC	17/25	1.00		
	TT+TC	177/240	1.17 (0.48–2.79)		
Ever	CC	27/42	1.00		
	TT+TC	388/306	2.05 (1.10–3.81)		
Drinking status					
Never	CC	19/37	1.00	0.915	0.178
	TT+TC	249/302	1.66 (0.78–3.53)		
Ever	CC	25/30	1.00		
	TT+TC	316/244	1.72 (0.87–3.39)		
Physical activity status				0.037	0.006
Inactive	CC	24/39	1.00		
	TT+TC	365/284	2.75 (1.39–5.41)		
Active	CC	20/28	1.00		
	TT+TC	200/262	0.89 (0.42–1.91)		
HBsAg status				0.373	0.263
Negative	CC	8/56	1.00		
	TT+TC	149/450	2.13 (0.98–4.63)		
Positive	CC	36/11	1.00		
	TT+TC	416/96	1.37 (0.66–2.84)		
HCC family history				0.187	0.064
No	CC	38/64	1.00		
	TT+TC	486/526	1.55 (0.93–2.59)		
Yes	CC	6/3	1.00		
	TT+TC	79/20	6.28 (0.90–44.04)		

Abbreviations: OR, odds ratio; 95% CI, 95% confidence interval.

^a^ORs and 95% CIs were calculated by logistic regression models after adjusting for age, gender, smoking and drinking status, physical activity, HBV infection status, and family history of HCC.

^b^*P* values for interaction were calculated by the multiplicative interaction term.

^c^*P* values for interaction were calculated using the additive scale in a bootstrapping procedure.

## DISCUSSION

The recognition that ARID1B exhibits tumor suppressor property has motivated growing interest in the research area of cancer etiology. This case-control study indicated that the predicted functional variant, rs73013281, significantly contributed to susceptibility of HCC. Particularly, the effect of the rs73013281T risk allele on HCC could be modified by physical activity.

ARID1B and its mutually exclusive isoform, ARID1A, are responsible for directing the target of the SWI/SNF complexes to certain promoters and consequently facilitate the control of gene expression programs [10]. Theoretically, the central function of the complexes in gene regulation, especially as anti-oncogene activator and oncogene repressor, could be altered by the ARID-subunits. There has been evidence showing aberrations in *ARID1B* leading to deletion of its protein-binding domain impaired the ability of SWI/SNF in induction of *TP53* and *P21* [9, 13], and also weakened the repression of Wnt/β-catenin signaling [14]. ARID1B also has profound impact on the feature of SWI/SNF in maintaining genome stability and cellular resistance to DNA damage, because it is required for the immediate recruitment of the ATPase in the complexes to DNA damage sites [20]. The experiments in liver cells has illustrated that the suppression of *ARID1B* would lead to deficient DNA repair and genomic instability that ultimately predisposed cells to malignant transformation [21]. Genome-wide co-activation analysis has also revealed the complexes involved in hepatic lipid metabolism through activating fatty acid oxidation genes, while the aberrations in the subunits would cause deregulation of fatty acid β-oxidation [22, 23], which has been linked to liver carcinogenesis [24]. Additionally, the components of SWI/SNF play important roles in immune response, and their alternations could make cancer cells invisible to the immune system [25]. Furthermore, *ARID1B* has emerged as tumor suppressor, for its frequent mutations have been recently identified across various cancers [2, 5, 7, 26].

In this study, the variant in *ARID1B*, rs73013281, was associated with HCC risk, showing a 74% higher risk for the CT/TT genotypes compared to the CC genotype (T allele frequency in controls = 0.689). Similarly, a previous study also showed the CT/TT genotypes of rs73013281 significantly increased the risk of breast cancer in the Moroccan population (T allele frequency in controls = 0.460) [27]. Nevertheless, the biological function of this variant has not yet been reported. As indicated by the function annotation from bioinformatics tools, the rs73013281, located 838bp 5′ from the start of *ARID1B*, was within a putative evolutionarily constrained region with promoter and enhancer histone marks, DNase hypersensitivity sites, and protein-binding sites in multiple tissues (liver tissue, etc; Supplementary Table 2). Additionally, based on the position weight matrices, the rs73013281T allele possibly disrupted a match to the regulatory motif for the transcription factor, EBF_know2, whereas the C allele created this match. Taken together, the T allele of rs73013281 may reduce the *ARID1B* expression by disrupting the affinity of certain transcription factors to the gene promoter or enhancer region, and consequently promote the genetic susceptibility to HCC.

More significantly, this study provides further evidence to support the interaction between genes and lifestyles in alteration of risk for complex diseases. In our results, among inactive individuals, the CT/TT genotypes of rs73013281 conferred a 2.75-fold risk of HCC compared to the CC genotype, whereas the CT/TT genotypes did not present association with HCC risk among individuals who exercise regularly, suggesting the rs73013281 may interplay with physical activity in modulation of HCC susceptibility. Although this gene-environment interaction has not been investigated until this study, some attempts still can be made based on available evidence. Physical activity has been indicated to lower the risk of hepato-carcinogenesis [28, 29], consistent with our results. The underlying mechanism possibly involves in changes of lipid metabolism, regulation of immune function, and activation of tumor suppressor [30]. As mentioned above, the SWI/SNF complexes also play important role in these processes. Therefore, it is held theoretically reasonable that the detrimental genetic effect of the rs73013281T allele could be attenuated by active lifestyle for HCC, emphasizing the importance of physical activity in prevention of HCC, particularly in those with genetic predisposition.

To our knowledge, it is the first investigation of the *ARID1B* variants in the genetic etiology of HCC. Nevertheless, it should be noted that potential selection bias might occur due to the hospital-based design. Due to the moderate sample size, the power to explore gene-environment interaction was still limited, and for further studies larger samples should be considered to fully deep potential interaction effect. Only three common variants were assessed, and this study did not provide a globe view to the genetic component of *ARID1B* in development of HCC. Additionally, we only collected information about whether subjects practiced exercise regularly each week in this study. A more precise analysis with exercise amount data is warranted to appraise the dose-response interactions between *ARID1B* variants and physical activity on HCC onset.

In summary, this case-control study suggested the potentially functional variant in *ARID1B*, rs73013281, as a new genetic susceptibility factor for hepatocellular carcinoma (HCC), especially in interaction with physical activity. These findings, highlighting the etiological role of the *ARID1B* variants in HCC development, may have prevention importance through identification of HCC-risk population as well as early detection of cancer, once these statistical associations are validated in large sample studies and functional biochemical experiments.

## MATERIALS AND METHODS

### Study subjects

The protocol of this study was approved by the institutional review board of Guangdong Pharmaceutical University. A total of 611 newly diagnosed HCC cases and 614 cancer-free controls were enrolled in this study. The subjects’ enrollment has been described previously [31]. In Brief, the HCC patients were confirmed by pathological examination or α-fetoprotein elevation (> 400 ng/ml) combined with imaging examination (computed tomography or magnetic resonance imaging) in Shunde First People's Hospital, Guangdong, China, during September 2010 and October 2014. During the same period of case enrollment, the cancer-free controls, frequency matched to cases by age (± 5 years) and gender, were randomly selected from a healthy screening at the same hospital. All subjects were unrelated Han ethnical residents in Shunde region. At recruitment, written informed consent has been obtained for each subject. The information about serological markers including HBsAg, anti-HBs, anti-HBc, and anti-HCV was collected by reviewing the medical records. Subjects positive for anti-HCV antibody were excluded in this study. In-person interviews were carried out by trained interviewers for all subjects to elicit epidemiological information, including gender, age (age at diagnosis for cases), smoking and drinking status, non-occupational physical activity, and family history of HCC in the first-degree relatives. The definitions of smokers, alcohol drinkers, and family history of HCC were described elsewhere [31]. Information about non-occupational physical activity for the last 10 years was collected by the question regarding whether subjects practiced exercise at least once per week. The active individuals were defined as those practice exercise at least once per week, while the subjects who exercise less frequent than once per week or with no leisure time activity were classified as inactive individuals. The response rate for cases and controls was 100%.

### Variants selection and genotyping

The potentially functional variants in *ARID1B*, with minor allele frequency > 0.05 in Han Chinese South or in Beijing (1000 Genomes Project: http://www.1000genomes.org), were searched using bioinformatics tools. For non-coding variants, HaploReg V4.1 tool (http://www.broadinstitute.org/mammals/haploreg/haploreg.php) was used to scan their regulatory effects [18]. The HaploReg V4.1 systematically explores functional annotations of the non-coding variants, incorporating chromatin mark ChIP-seq tracks and DNase tracks from the Roadmap Epigenomics Project, constrained sequence by SiPhy and GERP, and regulatory protein binding sites from the ENCODE ChIP-Seq data. For coding variants, PolyPhen-2 (http://genetics.bwh.harvard.edu/pph2/) was utilized to predict impact of an amino acid substitution on protein function via structural and comparative evolutionary considerations [19]. A total of three common variants, including rs73013281, rs167007, and rs9397984, were finally selected in this study based on their potential effects on regulatory motifs (Supplementary Table 2).

After the in-person interviews, 5-ml peripheral blood sample was collected from each subject. Genomic DNA was isolated using the TIANamp DNA kit (Tiangen, Beijing, China) according to the manufacturer's protocol. All of these variants were genotyped using the SEQUENOM MassARRAY iPLEX system according to the instructions of the manufacturer. Briefly, primers were designed by AssayDesigner software V3.1, and subsequent polymerase chain reaction (PCR) was performed. After single-base extension, the desalted products by SpectroCLEAN resin were spotted in a 384-format SpectroCHIP. Alleles was determined by the MassARRAY Analyzer Compact MALDI-TOF mass spectrometer. Data management was performed by Sequenom Typer V4.0. For quality control, genotyping was performed without knowledge of subjects’ disease status, and 5% samples were randomly selected for repeated assays, with concordance rate of 100%. The call rates were 99.8%, 99.7%, and 99.7% for rs73013281, rs167007, and rs9397984, respectively.

### Statistical analysis

The distribution of descriptive characteristics and genotypes were compared between cases and controls using *t*-test or Pearson's χ^2^ test, where appropriate. Hardy-Weinberg equilibrium for genotypes in controls was examined using the goodness-of-fit χ^2^ test. Association between the *ARID1B* variants and HCC risk were estimated using the adjusted odds ratio (ORs) and their 95% confidence intervals (CIs), which were calculated in logistic regression models with adjustment for age, gender, smoking, drinking and HBV infection status, physical activity, and family history of HCC. The plausible genetic model was assessed using the method as previously described [6, 32]. To reduce the potential spurious findings by multiple testing, a permutation method with 10,000 repeat was applied to calculate the empirical distribution of the *P* values observed for the genetic models of all variants [33]. The estimated statistical powers were 0.92, 0.55, and 0.50 for rs73013281, rs167007, and rs9397984, respectively, to detect the OR of 1.50. Haplotypes comprising these variants were constructed by Phase V2.1. Potential pair-wise interaction between the variants and selected variables were estimated using the multiplicative and additive scales. The *P* values for multiplicative interaction were calculated using the interaction term included in the multivariate logistic regression models, while the *P* values for additive interaction were estimated using a bootstrapping procedure that test the goodness-of-fit of the null hypothesis for no departure from an additive model against the alternative hypothesis. A two-tailed *P* < 0.05 was applied as the criterion of statistical significance. The permutation tests were performed using the software R V3.0.1, and other statistical analyses were conducted by the software SPSS V20.0.

## SUPPLEMENTARY MATERIALS TABLES


