# Supplementary material for: A potentially functional variant of ARID1B interacts with physical activity in association with risk of hepatocellular carcinoma
Source: Oncotarget. 2017 Mar 10;8(19):31057–64. doi: 10.18632/oncotarget.16074 (PMC5458188; doi:10.18632/oncotarget.16074)
Supplement: Supplementary file 1 [file oncotarget-08-31057-s001.pdf]

A potentially functional variant of *ARID1B* interacts with physical activity in association with risk of hepatocellular carcinoma

Supplementary Materials

Supplementary Table 1: The association between the variables and HCC risk

Variables	Crude OR (95% CI)	*P*	Adjusted OR (95% CI)[a]	*P*[a]
Smoking status				
Never	1.00		1.00	
Ever	1.64 (1.30–2.08)	< 0.001	1.54 (1.08–2.19)	0.011
Drinking status				
Never	1.00		1.00	
Ever	1.58 (1.26–1.98)	< 0.001	1.52 (1.11–2.08)	0.002
Physical activity status				
Inactive	1.00		1.00	
Active	0.63 (0.50–0.79)	< 0.001	0.68 (0.51–0.91)	0.006
HBsAg status				
Negative	1.00		1.00	
Positive	13.70 (10.39–18.07)	< 0.001	14.48 (10.77–19.47)	< 0.001
HCC family history				
No	1.00		1.00	
Yes	4.15 (2.58–6.68)	< 0.001	2.99 (1.71–5.23)	< 0.001

Abbreviations: HCC, hepatocellular carcinoma; OR, odds ratio; 95% CI, 95% confidence interval.
[a]ORs, 95% CI, and *P* values were calculated by the multivariate logistic regression models after adjusting for sex, age, smoking and drinking status, physical activity, HBsAg status, family history of HCC.

Supplementary Table 2: The functional annotation information for the genetic variants in *ARID1B*

Genetic variants	Alleles	Location in gene	MAF in CHB	*P* for HWE	Functional annotations					
					Conversation	Promoter histone marks	Enhancer histone marks	DNAse	Proteins bound	Motifs changed
rs73013281	C/T	5' near gene	0.175	0.101	Yes	24 tissue	14 tissues	52 tissues	4 bound proteins	EBF-known2
rs167007	A/G	Intron	0.070	0.952	No	--	3 tissue	--	--	NRSF-disc5
rs9397984	C/T	Intron	0.075	0.872	No	--	7 tissues	--	--	5 altered motifs

Abbreviations: CHB, Chinese Han population in Beijing; HWE, Hardy-Weinberg equilibrium; MAF, minor allele frequency.

Supplementary Table 3: Genetic association between the haplotypes of three variants in *ARID1B* and HCC risk

Haplotypes[a]	Cases (%) *N* = 1222	Controls (%) *N* = 1228	χ^2	*P*	OR (95%CI)
TGT	797 (65.2)	766 (62.4)	3.58	0.310	1.00
CGT	329 (26.9)	367 (29.9)			0.86 (0.72–1.03)
TAC	78 (6.4)	82 (6.7)			0.91 (0.66–1.27)
others	18 (1.5)	13 (1.1)			—

Abbreviations: HCC, hepatocellular carcinoma; OR, odds ratio; 95% CI, 95% confidence interval.

[a]The Haplotypes were constructed from the genotypes data of the variants arranged in the order of rs73013281, rs167007, and rs9397984.
